# A novel pulley–vest model enables progressive resistance exercise in rats without tail loading

**DOI:** 10.1007/s10974-026-09735-0

**Published:** 2026-07-14

**Authors:** Ronaldo André Castelo dos Santos de Almeida, Jéssica da Silva Santos, Gabriel Souza de Jesus, Letícia de Sousa Amorin, Ana Carolina Pereira da Silva, Hugo Adriano Oliveira de Sá Viana, Raphael da Silva Lau, Antônio Vicente Conrado Leite José da Costa, Fernando de Azevedo Cruz Seara, Anderson Luiz Bezerra da Silveira, Emerson Lopes Olivares

**Affiliations:** 1 Laboratory of Cardiovascular Physiology and Pharmacology, Department of Physiological Sciences, Institute of Biological and Health Sciences, Federal Rural University of Rio de Janeiro, BR 465, km 47, Seropédica, Rio de Janeiro CEP 23890-000 Brazil; 2 Laboratory of Physiology and Human Performance, Department of Physical Education and Sports, Institute of Education, Federal Rural University of Rio de Janeiro, Seropédica, RJ Brazil; 3 Department of Mathematics, Institute of Exact Sciences, Federal Rural University of Rio de Janeiro, Seropédica, RJ Brazil; 4 Center for Research in Precision Medicine, Carlos Chagas Filho Institute of Biophysics, Federal University of Rio de Janeiro, Rio de Janeiro, Brazil

**Keywords:** Strength training, Muscle hypertrophy, Dynamic performance, Pulley-Vest Training Model

## Abstract

Resistance training in rodent models is widely used to investigate muscular and cardiovascular adaptations. Traditional models often attach loads to the tail. The present study aimed to develop and validate a resistance training model for rats using a pulley system combined with a load-bearing vest, allowing progressive overload without tail attachment. Spontaneously hypertensive rats were assigned to control (CTR, *n* = 4) or resistance training (RT, *n* = 6) groups for a 10-week ladder climbing protocol. Functional and morphometric adaptations were evaluated in the soleus and flexor hallucis longus (FHL) muscles. Resistance training resulted in a significant increase in relative maximum load carried (*p* = 0.0007), corresponding to a 79.5% improvement in strength capacity. The RT group also showed increased relative mass of the FHL muscle (*p* = 0.002). Gene expression analyses did not show statistically significant differences between groups (*p* > 0.05). The pulley–vest system proved to be a viable and effective resistance training model for rats, allowing controlled overload while avoiding tail load attachment and potential vascular interference. This model represents a refined experimental approach for resistance training studies in rodent models, particularly in conditions where tail circulation must be preserved.

## Introduction

Resistance training is widely studied in sport science (Dias et al. 2024), nutrition (Aparicio et al. 2011), and medicine (Soares et al. 2022). Limitations on the participation of humans in invasive studies lead to the use of animal models to study the effects of resistance training (RT). The application of RT in animals is often limited by experimental procedures. Training animals to perform weight-bearing exercises depends on teaching these animals to perform the proposed movement and, in addition, training these animals to perform the movement with overload. A widely used method is climbing stairs with an overload attached to the animal’s tail (Al-Sarraf and Mouihate 2022; Cardoso Cassilhas et al. 2013a; Hornberger and Farrar 2004; Neto et al. 2016; Neves et al. 2016). Several resistance exercise models have been developed for animal studies, including ladder climbing with loads attached to the tail, squat-training devices, electrically induced resistance exercise, and weighted wheel running models, each with specific advantages and limitations (Alway et al. 1989; Amano et al. 2020; Baar and Esser 1999; Hornberger and Farrar 2004; Koopmans et al. 2024; Łochynski et al. 2016; Luciano et al. 2017; Nicastro et al. 2012; Tamaki 1992, 1992).

In addition to studies targeting hypertrophy, resistance training has been shown to be effective in combating signs of arterial hypertension (HBP) (Soares et al. 2022). This application may be limited if we consider that in rodents, the tail serves as an important thermoregulatory mechanism via the lateral artery of the tail (Feng et al. 2008; Rand et al. 1965; Wilde et al. 2017), in addition to the circulation itself. In studies with spontaneously hypertensive animals (SHR), any vascular blockage can interfere with the interpretation of results. Although the ladder-climbing method is effective in training rats as experimental models (Krause Neto et al. 2024; Kwon 2025), the risk of attaching weights to the animals’ tails can be problematic due to interference with blood flow at the attachment site, as well as issues with the need for stimulation and aversive stimuli to encourage the animals to climb the ladder by pulling weights attached to their tails. (Al-Sarraf and Mouihate 2022). In this study, SHR were used because this model is intended for future application in cardiovascular disease studies. Since these animals already present vascular and hemodynamic impairment, they provide a more clinically relevant and methodologically demanding condition for validating a resistance training model that avoids tail compression.

Based on previous literature, ladder climbing appears to be the most widely used and well-established method for resistance exercise in rats, but remains still necessary to develop a model that is easier to apply and does not interfere with the blood circulation. An important limitation of this model is the need for some type of induction for the animal to climb with loads tied to its tail (Hornberger and Farrar 2004). Our study demonstrates the application of resistance training on an inclined ladder using a vest to fix the loads, leaving the animal’s tail without any possibility of arterial occlusion. Our study verified the validity of our model by comparing morphometric data and the progression of the maximum load supported in trained animals (RT).

Skeletal muscle exhibits marked plasticity in response to mechanical stimuli (Caiozzo 2002; Haun et al. 2019a). Repeated loading, such as resistance exercise, can promote neuromuscular, structural, metabolic, and molecular adaptations, whereas unloading or reduced mechanical demand may lead to muscle atrophy and impaired function (Nunes et al. 2022). These adaptations depend on the magnitude, frequency, and progression of the mechanical stimulus, highlighting the importance of experimental models that allow controlled overload, reproducible movement patterns, and minimal interference from the apparatus itself. Therefore, refined rodent models that permit progressive resistance exercise while reducing potential confounding factors are important tools for investigating skeletal muscle adaptation.

Therefore, the objective of the present study was to evaluate the feasibility and validity of a resistance training model using an inclined ladder with a load-bearing vest, allowing the application of overload without attaching weights to the tail and minimizing potential occlusion of blood circulation.

## Materials and methods

The animal study was reviewed and approved by the Animal Ethics Committee – CEUA 14/2022/UFRRJ, always prioritizing the 3Rs principle.

### Animals

SHR (male, 105 days old, 252.1 *±* 9.9 g) were obtained from the Central Bioterium of the Health Sciences Center of the Federal University of Espírito Santo. The animals were kept at an ambient temperature of 23 (*±* 1^°^C) under a 12 h light-dark cycle (lights on from 6:00 a.m. to 5:59 p.m.) with free access to pelleted chow and water. Rats were randomly divided into a control group (CTR, no exercise, *n* = 4) and a resistance exercise group (RT, 10 weeks, *n* = 6). Since the trained group was the central group for validating the new exercise model, a greater number of animals was initially maintained in this group to ensure adequate evaluation of training performance, adaptation to the apparatus, and progression of overload across the protocol. Thus, the slightly larger RT group was used to strengthen the assessment of the feasibility and reproducibility of the intervention.

After the habituation period of 7 days, baseline performance was assessed. Subsequently, a 10-week progressive resistance training program was performed 3 times per week. Rats were weighed (Toledo Prix 3, Toledo, Brazil) pre- and post-training period in this study. After 10 weeks, rats were euthanized and samples of muscles of the hind limb (soleus and FHL), which are most involved during this resistance exercise, were harvested, weighed on a microbalance (AD 500, Marte, Brazil), and then were either snap frozen in liquid nitrogen and stored in − 80^°^C freezer for later PCR analysis.

### Resistance exercise apparatus

**Fig. 1:**
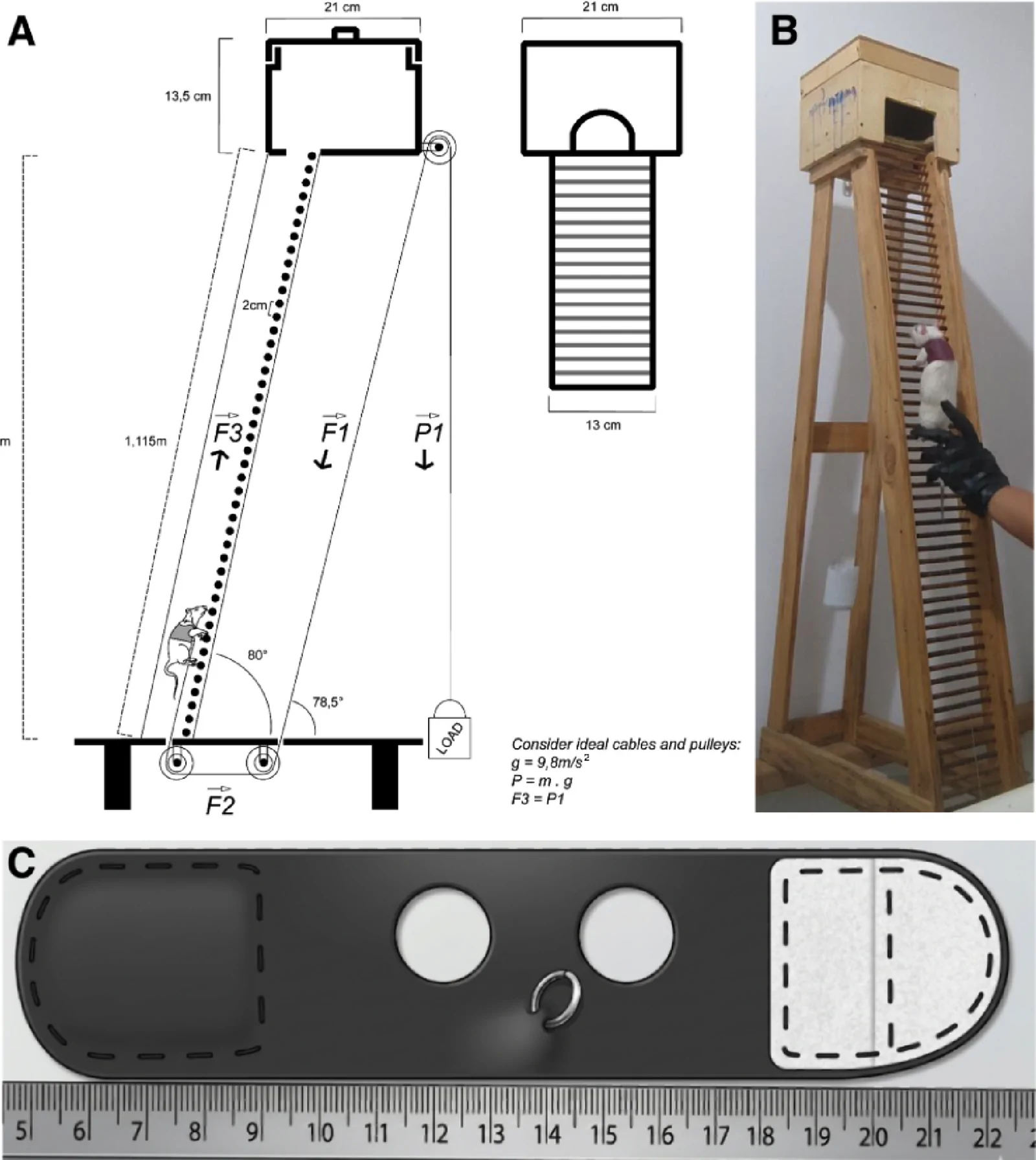
Training apparatus. (**A**) Schematic representation of the resistance training apparatus used for ladder-climbing exercise in rats. The external load is applied through a cable-and-pulley mechanism connected to a suspended weight (P1), which generates a traction force (F3) transmitted along the climbing direction. The mechanical relationship was considered under ideal conditions, assuming negligible cable mass and pulley friction, with the pulley changing only the direction of the applied force without providing mechanical advantage. The traction force transmitted to the animal corresponds to the weight of the suspended load (g = 9.8 m/s²; P1 = m·g; F3 = P1). (**B**) Representative image of a rat undergoing training on the apparatus. (**C**) Structural design of the weighted vest used for resistance training in rats. The metallic ring positioned in the ventral midline serves as the attachment point for the external load during exercise. A ruler is shown for scale (centimeters)

The resistance exercise apparatus shown in Fig. 1A was designed and built in our laboratory. This apparatus consists of a ladder with an extension of 1,115 m and a housing chamber at the top with an entrance hole at the base. The animal was trained to climb the ladder by pulling a load attached to the vest. At the end of the climb, the animal recovered between climbs in the housing chamber. The ladder was mounted at an 80° angle from the base. The crossbars of the ladder have a diameter of 4 mm, and their axes are spaced 10 mm apart. We utilized 55 steps to improve muscle endurance. These adjustments allow the stairs to accommodate rats of various sizes. Compared to a model where the load is dragged from the tail, our pulley system preserves the original overload weight, considering ideal cables and pulleys.

**Fig. 2:**
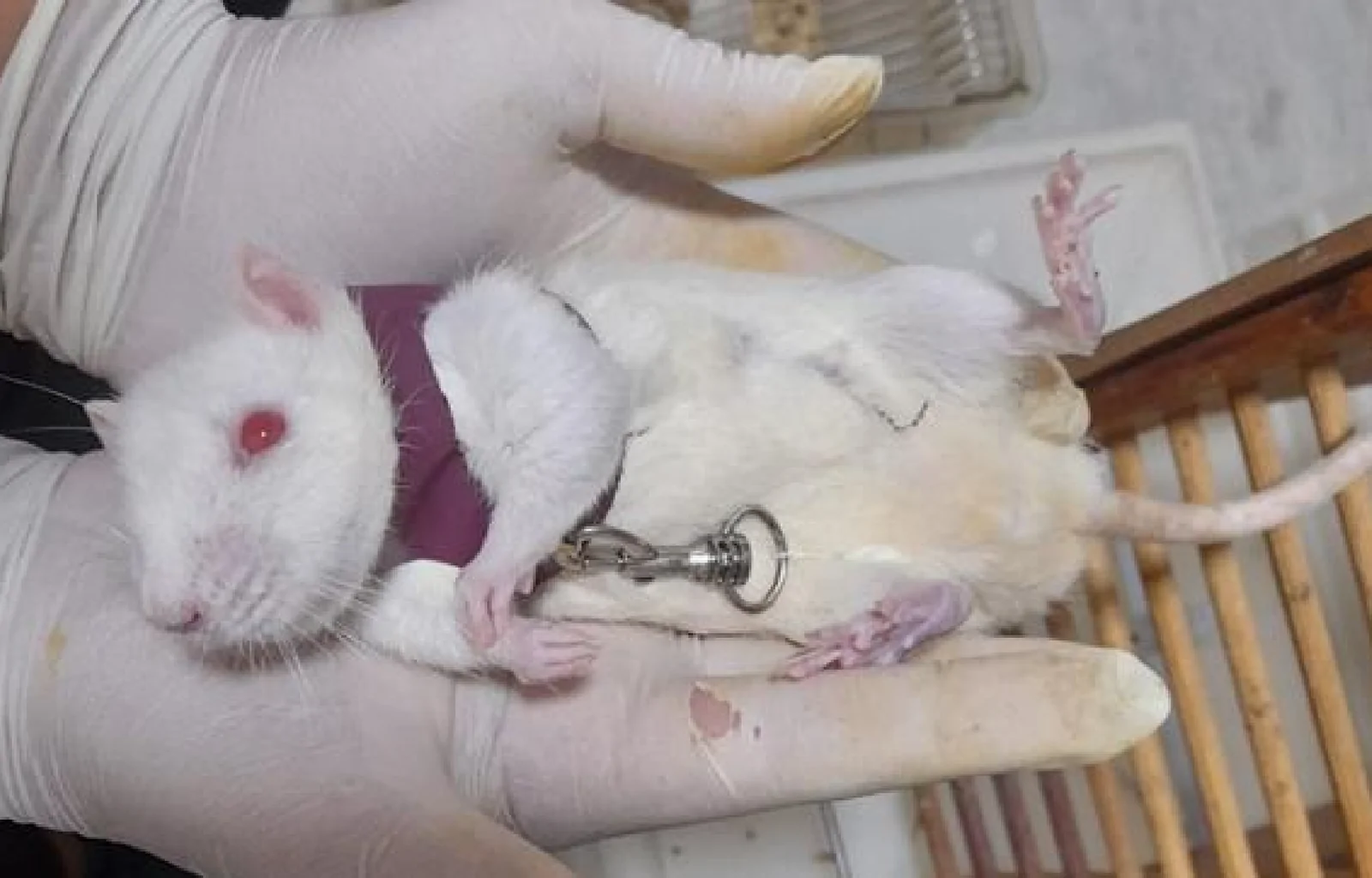
A rat equipped with a training vest used for applying overload during resistance exercise. The vest, fitted to the animal’s body, has a metal attachment point connected to a pulley system for weight traction during ascent of the training ladder

We designed a custom-weighted vest to allow for the application of external load during stair climbing exercises, avoiding the need to attach weights to the animal’s tail (Fig. 1C). The vest was made from a flexible cotton and elastane fabric, providing elasticity, comfort, and a proper fit to the animal’s body. The material was cut to form a vest-like structure, with the front opening allowing positioning around the neck, while the back portion encompassed the thoracic region. Lateral openings were created to allow unrestricted movement of the forelimbs during climbing, similar to a previously used model (Hinks et al. 2022). A Velcro fastening system was incorporated into the dorsal region of the vest to allow for secure attachment and quick adjustment. The additional load was attached to the vest via a cable anchored to a piercing positioned on the ventral side. The working load was attached to the other end of the cable. This design allowed for progressive load increases throughout the training protocol in a simple and quick way, maintaining normal mobility and avoiding interference with blood circulation in the tail or thermoregulatory function. The animals fit comfortably in the vest and, over the course of training sessions, become fully adapted to handling it (Fig. 2).

### Adaptation to the training apparatus

The animals underwent a 7-day adaptation protocol before the start of training to reduce the risk of interference in performance. Adaptation followed the procedure shown in Table 1.

**Table 1:** Protocol for adapting animals to the training apparatus. In addition to the specific movement of the exercise, the animals also underwent periods of adaptation to the housing chamber at the top of the stairs

Day	Procedure
1	10 min at housing chamber 10 climbing with no load WITHOUT vest / Free interval
2	10 min at housing chamber 10 climbing with no load WITHOUT vest / Free interval
3	10 climbing with no load WITH vest / Free interval 10 min at housing chamber
4	3 climbing WITHOUT load WITH vest + 7 WITH 10 g load and vest / Free interval 10 min at housing chamber
5	3 climbing WITHOUT load WITH vest + 7 WITH 10 g load and vest / Free interval 10 min at housing chamber
6	3 climbing WITHOUT load WITH vest + 7 climbing WITH 20 g load and vest / 2–3 min interval
7	3 climbing WITHOUT load WITH vest + 7 climbing WITH 30 g load and vest / 2–3 min interval

### Maximum Load Carried Test (MLCT)

The MLCT was conducted according to Table 2. A 2-minute rest interval was proposed between each attempt, as recommended by Hornberger and Farrar (Hornberger and Farrar 2004) for maximum strength tests. The test was interrupted when the rat failed or refused to climb. If, after 3 attempts, the animal demonstrated the condition to continue climbing, the test was repeated after 6 h or the following day, starting with the last load performed.

**Table 2:** Overload in the maximum transported load test

Attempt	Load
1	50% BW
2	+ 10% of the first attempt
3	+ 5–10% of the second attempt

BW: Body weight

### Resistance training procedure

Training followed the progression shown in Table 3. The total height of the climb was 1,115 m. According to a previous protocol (Neves et al. 2016), the initial weight fixed on the tail was 30% of the MLCT. Each training session consisted of one set with 1 (approximate time) minute of rest between attempts. After the fifteenth session, the weight was readjusted with a new MLCT. After the last day of training, all rats performed the MLCT again.

**Table 3:** Training protocol adopted in this study. Progression of the number of climbs per week and the load in the sessions

Sessions	Week	Ladder climbs	% Load MLCT
1–3	1	1 to 3 4 to 6	30 50
4–9	2–3	1 to 2 3 to 6 7	30 50 60
10–30	4–10	1 to 2 3 to 4 5 to 6 7 to 8	30 50 60 70

MLCT: Maximal load carried test. Training sessions were interrupted if the animal did not conclude a climb

### Tibia length measurement

Tibia length was measured as an indicator of body size and skeletal growth, as commonly used in rodent studies to normalize muscle mass and morphological parameters. After euthanasia, the tibia was carefully dissected and cleaned of surrounding soft tissue. The length of the tibia was measured using a digital caliper (Precision of 0.01 mm, model 500-196-20B, Mitutoyo, Betim, Minas Gerais, Brazil) from the proximal tibial plateau to the distal medial malleolus. This parameter was used as an index of body size and to assist in the interpretation of muscle mass data.

### Euthanasia

The rats were euthanized by decapitation. Immediately after decapitation, death was confirmed by the absence of respiratory movements and reflexes. This method was adopted to allow for parallel ex-vivo analyses of the hearts in another exploratory study. Skeletal muscles were collected immediately afterward. All tissue samples were collected immediately after euthanasia to minimize post-mortem degradation and preserve tissue integrity for molecular analyses, and were frozen in liquid nitrogen and then stored at −80.

### Polymerase Chain Reaction (PCR)

RNA extraction was performed using approximately 30 mg of tissue collected from the cardiac apex of the animals, using the RNeasy^®^ Fibrous Tissue Mini Kit (QIAGEN^®^), according to the manufacturer’s recommendations. Initially, the tissue fragments were homogenized in 300 µL of RLT buffer supplemented with β-mercaptoethanol using a rotary homogenizer. Then, 590 µL of RNase-free water and 10 µL of proteinase K were added, and the samples were incubated at 55 °C for 10 min. Subsequently, they were centrifuged at 25 °C for 3 min at 10,000 × g. Absolute ethanol (100%) was added to the supernatant obtained in a 0.5:1 (v/v) ratio, and 700 µL of the mixture was transferred to purification columns coupled to 2 mL microtubes. After centrifugation at 8000 × g for 15 s at 25 °C, the eluate was discarded, and this step was repeated to ensure better RNA binding to the column membrane. To remove contaminants, 350 µL of RW1 buffer was added to the columns, followed by centrifugation under the same conditions. Enzymatic digestion of genomic DNA was performed with 80 µL of the mixture containing 10 µL of DNase I and 70 µL of RDD buffer, incubating at room temperature for 15 min. Next, the washing step with RW1 buffer was repeated, followed by two washes with 500 µL of RPE buffer, the last one with centrifugation for 2 min at 8000 × g. For the final RNA elution, the columns were transferred to 1.5 mL tubes, and RNase-free water was added. The elution process was carried out with two consecutive centrifugations at 8000 × g for 1 min at a temperature of 25 °C, ensuring maximum nucleic acid recovery.

To estimate mRNA expression levels of E3 ubiquitin-protein ligase (Trim63), F-box only protein 32 (Fbxo32), and myogenic factor 6 (Myf6) (Table 4), total RNA was extracted from skeletal muscle tissue samples using the RNeasy^®^ Fibrous kit. Tissue Mini Kit (QIAGEN) and cDNA was prepared from 1 µg of total RNA using the High-Capacity Reverse Transcription kit (Thermo Fisher Scientific) according to the manufacturer’s instructions. mRNA levels of target genes (Table 4) were evaluated by qRT-PCR. Amplification reactions containing 1ng of cDNA were performed at 60 °C during the annealing and extension cycles. The expression of chosen genes was normalized to GAPDH as an internal control. The quantification of selected mRNA was determined by 2-(ΔΔCT) method in a QuantStudioTM Design e Analysis V 1.5.2 and expressed as fold change of RT compared to the CTR.

**Table 4:** Description of the nucleic acid sequence of the primers used in the study

Primers	Forward	Reverse
GAPDH	GCCTTCCGTGTTCCTACC	CCTCAGTGTAGCCCAAGATG
Trim63	CCCCTTACAAAGCATCTTCCA	TGTTTTCCTTGGTCACTCGG
Fbxo32	CAACAGACTGGACTTCTCGAC	GAAGTTCTTTTGGGCGATGC
Myf6	TTTGAGTCCATCACCCAGTTC	GCCCTCTGCCACTTCTAATG

For gene expression analysis, *n* = 4 per group was used because only samples with sufficient tissue availability and RNA quality were included in the molecular assays. In addition, because the molecular analysis was considered exploratory and complementary to the main functional outcomes of the study, we restricted the PCR analysis to biologically representative samples that met the predefined technical quality criteria.

### Statistical analysis

Because of the small sample size, data distribution was carefully inspected before inferential testing. For between-group comparisons, the Mann–Whitney test was used when normality or variance assumptions could not be confidently supported. For the pre-post intragroup comparison, the paired t-test was used. When parametric assumptions were satisfied, Student’s t-test was retained. Results are presented as mean ± SEM and individual values, with statistical significance set at *p* < 0.05. Effect sizes were calculated and reported as eta squared (η²). All statistical analyses and graphical representations were performed using GraphPad Prism version 10.1.1 (270) (GraphPad Software, San Diego, CA, USA).

## Results

### Body weight gain

**Fig. 3:**
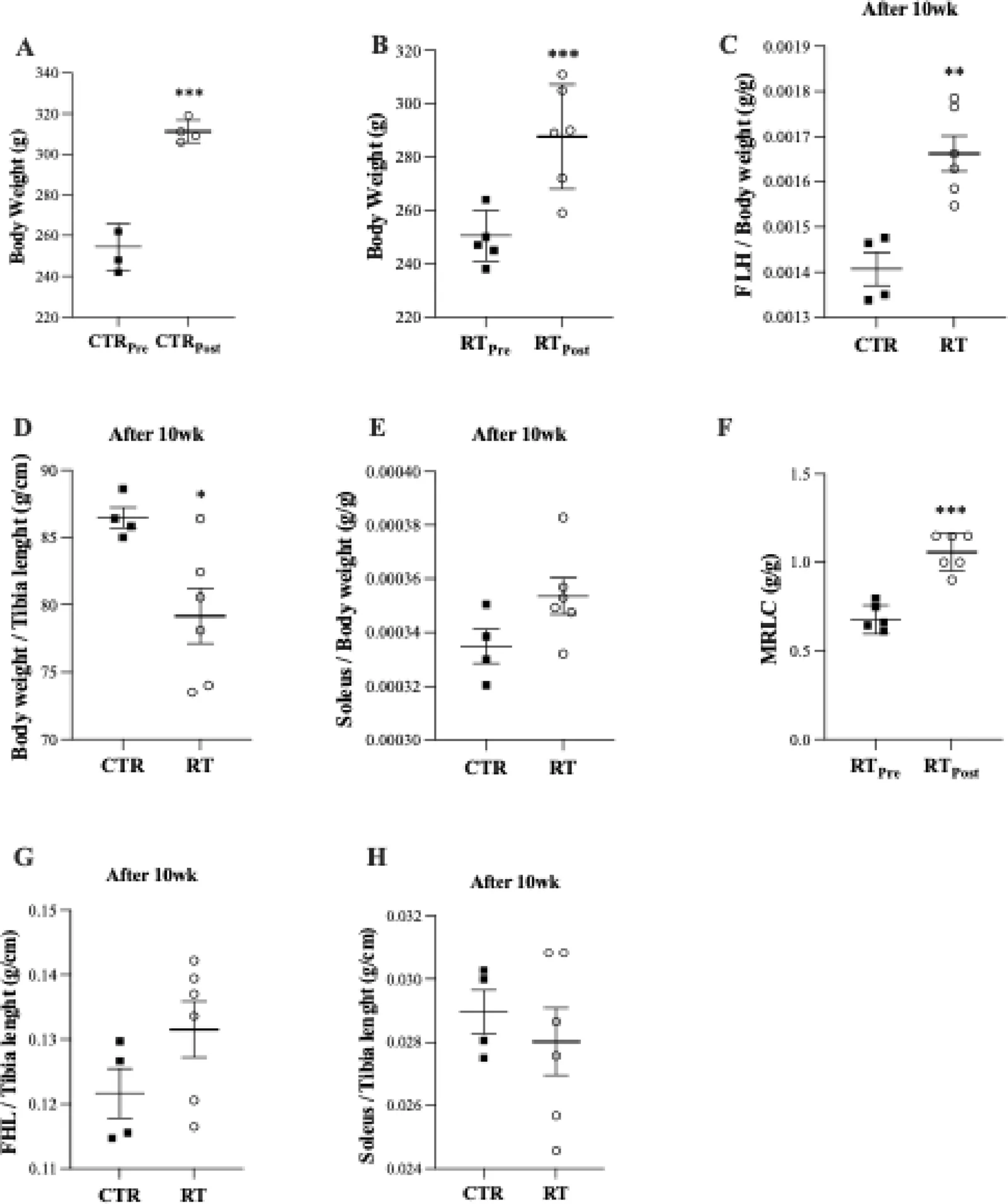
(**A**) Body weight of CTR (*n* = 4), (**B**) Body weight of RT (*n* = 6), (**C**) Relative body weight after 10wk (Body weight / tibia length), (**D**) Relative FHL weight (FHL weight / body weight), (**E**) Relative soleus weight (Soleus weight / body weight), (**F**) Maximum relative load carried (MRLC). (**G**) FHL weight / tibia lenght. (**H**) Soleus weight / tibia lenght. The data are presented individually with mean ± SEM. A paired t-test, two-tailed, was used to compare the groups at the pre and post time points. Student’s t-test, Two-tailed, was used for comparisons between groups. **p < 0,01; ***p < 0,001

**Fig. 4:**
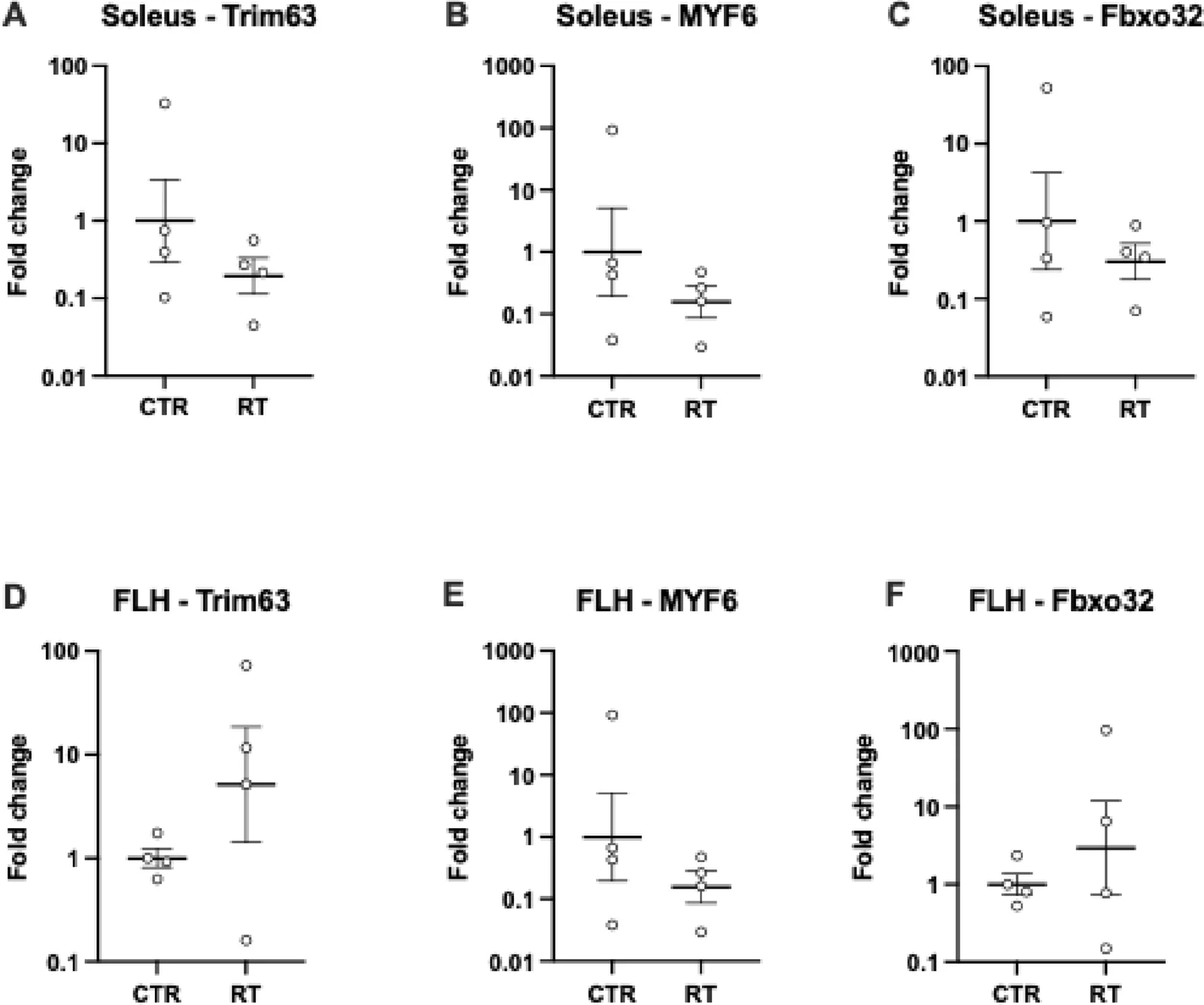
Relative gene expression of Trim63, Fbxo32, and MYF6 in soleus (predominantly oxidative) and FLH (Flexor Longus Hallucis, predominantly glycolytic) muscles in control (CTR, *n* = 4) and trained (RT, *n* = 4) groups. Expression was normalised to GAPDH and expressed as fold change relative to the control group. Data are presented individually with mean ± SEM. Student’s t-test (Two-tailed) was used for comparisons between groups

At the beginning of the study, the randomly assigned animals did not have different average body weights between the groups (Fig. 3A−B; *t* = 0.6, CI_95%_ −19.3 to 11.2, η² = 0.04, *p* > 0.05); CTR = 254.5 g (± 5.6), and RT = 250.5 g (± 3.8). On the day of euthanasia, average body weights did not differ between the groups (Fig. 4A−B; *t* = 2.3, CI_95%_ −47.2 to 0.02, η² = 0.4, *p* > 0.05); CTR = 311.3 g (± 2.7 g), and RT = 287.7 g (± 7.9). CTR and RT groups had a body weight gain of 22,3% and 14,8%, respectively, at the end of 10 weeks compared to their weight at the beginning of 10 weeks (*p* < 0.001). When body mass was normalized for tibia length (Fig. 3C), RT showed lower mass compared to CTR (*t* = 2.8, CI_95%_ −13.3 to −1.2, η² = 0.5, *p* = 0.02).

### Muscle weights

Figure 3D−E shows the wet weights for the hind limb muscles in the RT group compared to their corresponding muscles in the CTR. No difference was observed in the mass of the soleus muscle normalized by body mass (*Mann-Whitney U* = 4, CI_95%_ −2.941 × 10⁻⁶ to 4.420 × 10⁻⁵), rank-biserial correlation = 0.67, η² = 0.3, *p* > 0.11). RT had a higher relative mass of FHL compared to CTR (*Mann-Whitney U* = 0, CI _95%_ 1.088*10^− 4^ to 4.279*10^− 4^, rank-biserial correlation = 1.00, η² = 0.7, *p* = 0.0095)

### Maximum Relative Load Carried (MRLC)

Relative load progression increased significantly following the resistance training period, as shown in Fig. 3F. Paired comparisons between pre-training (RT_pre_) and post-training (RT_post_) conditions demonstrated a significant increase in relative load carried (*t* = 7.464, CI_95%_ 0.2 to 0.5, η² = 0.9, *p* = 0.0007). Resistance training provided a 79.5% increase in strength.

The magnitude of the observed effect was large (partial η² = 0.9176), indicating that the majority of the variance in relative load was associated with the training intervention. These findings demonstrate a robust progression in load tolerance over the training period, reflecting substantial improvements in the animal’s capacity to sustain higher relative loads after the resistance training protocol.

### PCR

In the soleus (Fig. 4A−C), the expression of Trim63 showed similar distributions between the CTR and RT groups (*t* = 1.2, CI_95%_ −2.1 to 0.7, η² = 0.2, *p* = 0.3), with overlapping medians and interquartile ranges, suggesting no clear effect of resistance training on its expression. A similar pattern was observed for MYF6 in the soleus muscle, where the CTR and RT groups showed similar expression levels, with little separation between the groups (*t* = 1.1, CI_95%_ −2.6 to 1, η² = 0.16, *p* = 0.3). The expression of Fbxo32 also showed no difference in RT compared to the CTR group (t = 0.8, 95% CI −2.1 to 1.1, η² = 0.09, *p* = 0.5), just the greater dispersion of individual values.

In the FHL (Fig. 4D−F), composed predominantly of fast-twitch fibers, we also observed no significant differences for Trim63 between the RT group compared to the CTR group (t = 1.3, 95% CI −0.7 to 2.1, η² = 0.2, *p* = 0.2), with a wider distribution of individual values for RT. For MYF6, there was no difference between the RT group and the CTR group (t = 1.1, 95% CI −2.6 to 1, η² = 0.16, *p* = 0.3). For Fbxo32 in the FHL, the RT group again showed no difference compared to the CTR group (t = 0.7, 95% CI −1.1 to 2, η² = 0.08, *p* = 0.5).

## Discussion

To our knowledge, this is the first report of an experimental resistance exercise model using a pulley-and-vest system for weight pulling in rats. To validate this experimental model, we tested the pulling strength as well as explored skeletal muscle hypertrophy through muscle weights. The resistance exercise increased by pulling strength and in relative FHL mass in the RT group. To evaluate muscle hypertrophy, we selected two ankle extensor muscles as ankle extension seems to be actively involved in the walking movement of the rats undergoing pulling against weights through the stairs. We did not observe significant hypertrophy in the soleus muscle, probably because this muscle has a predominantly slow-twitch oxidative phenotype and a high proportion of type I fibers (Júlio Chingui et al. 2008). Slow-twitch muscles such as the soleus are generally characterized by high oxidative capacity, postural function, and greater resistance to fatigue, but they may show a lower hypertrophic response to overload protocols compared with muscles containing a higher proportion of fast-twitch fibers. In contrast, the FHL muscle, which is more strongly recruited during loaded climbing and has a greater contribution of fast-twitch fibers, may be more susceptible to overload-induced increases in muscle mass. Therefore, the selective increase in FHL mass observed in the present study is consistent with the well-established fiber-type-dependent adaptability of skeletal muscle (Hornberger and Farrar 2004). This differential response may also reflect muscle-specific susceptibility to phenotype modulation under varying mechanical conditions, since skeletal muscle phenotype is dynamically regulated by loading and unloading stimuli. Thus, the absence of detectable soleus hypertrophy does not necessarily indicate a lack of adaptation, but rather suggests that the pulley–vest resistance training model preferentially induced morphometric adaptation in muscles more directly involved in force production during ladder climbing.

The observed increase in muscle mass (Fig. 3D), and the absence of alterations in gene expression related to athrophy, can be attributed to structural adaptations independent of transcriptional regulation, particularly to the increase in intramuscular glycogen content and the consequent retention of intracellular water (Haun et al. 2019b; Henselmans et al. 2026). Since glycogen storage is osmotically active, its accumulation promotes cell swelling, leading to expansion of muscle fiber volume (Henselmans et al. 2026; Shiose et al. 2023). Furthermore, hypertrophy can occur through sarcoplasmic expansion (Haun et al. 2019b; Roberts et al. 2020), characterized by an increase in cytoplasmic volume and metabolic components without a proportional increase in contractile proteins. These mechanisms contribute to measurable increases in muscle mass independent of the activation of classical gene expression pathways associated with myofibrillar hypertrophy.

We selected three genes for this study, Trim63, Fbxo32, or Myf6. The selected genes were chosen because they represent key regulatory pathways involved in skeletal muscle remodeling in response to resistance training, particularly the balance between protein synthesis, protein degradation, and myogenic regulation. Trim63 (MuRF1) and Fbxo32 (Atrogin-1) are muscle-specific E3 ubiquitin ligases and are widely recognized as central markers of the ubiquitin–proteasome system, the primary pathway responsible for myofibrillar protein degradation (Bodine and Baehr 2014). These genes are commonly used as molecular markers of muscle protein turnover and remodeling in response to mechanical loading and unloading. Myf6 (also known as MRF4) is a myogenic regulatory factor involved in muscle differentiation, maturation, and fiber-type remodeling, and has been associated with muscle adaptation and regeneration processes following resistance exercise (Schiaffino and Reggiani 2011).

Therefore, the selection of these genes allowed us to investigate whether the resistance training protocol induced molecular responses related to muscle protein degradation pathways (Trim63 and Fbxo32) and myogenic regulation (Myf6), providing insight into the mechanisms of muscle adaptation beyond morphometric changes.

Gene expression analysis did not reveal statistically significant differences between groups for Trim63, Fbxo32, or Myf6. Therefore, no definitive conclusions can be drawn regarding the molecular mechanisms of hypertrophy or atrophy. The molecular data presented here should be interpreted as exploratory and descriptive rather than mechanistic. The variability observed between muscles may suggest muscle-specific responses; however, these findings require confirmation in studies with larger sample sizes and additional protein-level analyses. Importantly, the primary objective of this study was not to investigate molecular pathways, but to validate a resistance training model capable of producing functional and morphometric adaptations without tail load fixation.

Another important consideration is the timing of molecular measurements. In the present study, muscle mass and gene expression were assessed only at the end of the 10-week protocol. Therefore, the absence of significant differences in Trim63, Fbxo32, and Myf6 expression at this endpoint does not exclude transient molecular responses occurring earlier during training. Gene expression related to protein turnover, myogenic regulation, or anabolic signaling may peak during the initial days or weeks of adaptation and return toward baseline by the end of the protocol. Future studies should include multiple time points and animals euthanized specifically during early and intermediate phases of training to better define the temporal dynamics of molecular adaptation.

Our proposed 10-week training protocol is close to the upper limit of the 6–12 week range that is observed in several studies (Al-Sarraf and Mouihate 2022; Cardoso Cassilhas et al. 2013a; Hornberger and Farrar 2004). A previous study reported a 23% increase in flexor halluces longus muscle mass after 8 weeks of exercise (Hornberger and Farrar 2004). Our model was based on the overload principle, and it was designed for the number of sets, repetitions, and rest periods between sets to closely resemble that of a typical RT program for humans. Despite this, we must consider the rapid structural development of rats, and this characteristic requires a greater amount of work than humans to achieve similar hypertrophic effects of the resistance exercise (Al-Sarraf and Mouihate 2022; Klitgaard 1988). Therefore, a greater frequency of training may be needed to induce muscle hypertrophy in rodents (Al-Sarraf and Mouihate 2022; Cardoso Cassilhas et al. 2013b; Strickland and Smith 2016).

The proportion of fast-twitch muscle fibers varies depending on the muscle (Schiaffino and Reggiani 2011). The load progression (Fig. 3F) corresponds to that observed in human strength studies (Kraemer and Ratamess 2004). In addition to the benefits specifically desired for combating HBP, our training protocol in this new model also produced strength gains and promoted increased muscle mass. These results suggest that our model was effective and can be used in studies of exercise and performance.

Maximum relative load carried (MRLC) increased progressively across the training sessions, indicating effective adaptation to the resistance training protocol. Animals were able to sustain progressively higher loads relative to their body mass throughout the training period. A gradual increase in MRLC from the initial sessions to the final stages of training reflects improvements in strength performance and tolerance to overload. Such progressive increases in load are consistent with adaptations expected in rodent resistance training models, in which repeated climbing bouts under increasing external load promote neuromuscular and muscular adaptations (Cardoso Cassilhas et al. 2013a; Hornberger and Farrar 2004).

It is important to consider, however, that laboratory rodents continue to undergo somatic growth and musculoskeletal maturation during experimental periods. In young or young-adult rats, increases in muscle mass and force-generating capacity may occur as part of normal development, due to processes such as fiber hypertrophy, neuromuscular maturation, and changes in muscle architecture (Armstrong and Phelps 1984; Schiaffino and Reggiani 2011). Consequently, part of the increase in load tolerance observed across training sessions may be partially influenced by the natural development of the animals.

Nevertheless, the progressive increase in MRLC relative to body mass across repeated training sessions suggests that the improvements observed were not solely attributable to growth-related changes. Previous studies using climbing-based resistance exercise models have demonstrated that progressive overload protocols induce specific neuromuscular and hypertrophic adaptations in rodents beyond those expected from normal maturation (Hornberger and Farrar 2004; Strickland and Smith 2016). Therefore, the pattern of load progression observed in the present study likely reflects both physiological development and training-induced adaptations associated with repeated exposure to the resistance exercise stimulus.

The increase in maximum relative load carried should not be interpreted as evidence that muscle hypertrophy alone occurred. Because the test was performed in the same apparatus used for training, improvements may reflect neural adaptation, motor learning, improved climbing skill, better coordination, and familiarization with the pulley–vest system, in addition to possible muscular adaptations. Thus, this outcome is best interpreted as improved load-carrying performance within the proposed model.

Since our model is for studying HBP in SHR, the loads used are lower than conventional hypertrophy protocols. This factor, associated with a longer adaptation protocol to the ladder, eliminated the need to use external stimuli to induce the animal, refining the method and contemplating the 3Rs principle. As described previously, the pulling stimulus on the tail of the rat seems to prompt an innate response to move forward (Al-Sarraf and Mouihate 2022). The development of the vest provides greater comfort to the animal after adaptation, as it does not block circulation in the lateral artery of the tail. We wondered whether the vest could be limiting the expansion of the rib cage and preventing the animals from performing movements. Our observations support the idea that this did not happen. During the adaptation period, if the animal stood on the base of the ladder, it was very common for it to get rid of the vest. To redouble care and avoid any damage, we suggest prioritizing malleable materials for making the vest. Although our model requires a period of training in the apparatus used, this learning eliminates the need for some type of aversive stimulus to drive the animal to perform the training. After the first days of adaptation, the rats showed no overt signs of stress, like droppings or vocalization.

We did not compare the results of our model with any other model available in the literature. The aim of this study was to develop and validate the feasibility of a new resistance training model, not to compare training methods. A direct comparison with tail-loading models is an important next step and has now been acknowledged as a limitation and a future research direction.

We used only male rats. Male animals were selected to minimize the variability associated with hormonal fluctuations related to the estrous cycle, which can influence muscle metabolism, performance, and gene expression (Haizlip et al. 2015; Liu et al. 2010). Although this approach is common in initial validation studies to improve experimental control, it limits the generalizability of the results. Future studies should evaluate the responses of female rats to this resistance training model, since sex-related differences in muscle adaptation and cardiovascular responses to exercise are well documented.

Despite the promising results obtained with the pulley vest resistance training model, some limitations of the present study should be considered. First, the sample size was relatively small. Future studies with larger samples may improve the robustness of the statistical analyses. Second, the present investigation focused primarily on functional and morphometric outcomes, such as progression of strength and muscle mass. Although the RT group showed higher FHL mass when normalized to body mass, this finding should be interpreted cautiously because body weight differed between groups. Since fiber cross-sectional area, histological characterization, and anabolic signaling pathways were not assessed, the present data do not allow us to conclude that true myofiber hypertrophy occurred. Therefore, the increase in relative FHL mass is interpreted as a morphometric adaptation supporting the feasibility of the model, rather than definitive evidence of hypertrophy.

The present analysis was restricted to the soleus and FHL muscles, which were selected because they represent muscles with different functional and fiber-type characteristics and are likely involved during ladder climbing. However, this limited sampling does not fully characterize the muscle-specific adaptations induced by the model. Future studies should include additional hindlimb muscles, including anterior compartment muscles such as the tibialis anterior and extensor digitorum longus, as well as thigh muscles, to determine whether the pulley–vest model induces broader or compartment-specific adaptations.”

The molecular analysis was restricted to selected atrophy-related genes, including Trim63 and Fbxo32, which do not directly represent hypertrophy-specific signaling pathways. The inclusion of molecular targets related to skeletal muscle hypertrophy and mechanotransduction, such as MEF2, SRF, YAP and other Hippo pathway-related genes, mTORC-related targets, and MSTN, would strengthen the mechanistic interpretation of the skeletal muscle adaptations observed in this model.

Another limitation concerns the duration and intensity of the training protocol. As the model was designed for use in spontaneously hypertensive rats, the applied loads were intentionally lower than those commonly used in hypertrophy-focused protocols. In studies aiming for this objective, we suggest that applying longer training periods or different loading strategies may help to better characterize the hypertrophic potential of the model.

A further limitation is the absence of a conventional tail-loading comparison group. Therefore, although the pulley–vest system avoids direct attachment of the load to the tail, the present study did not directly quantify tail blood flow, vascular occlusion, local injury, or stress responses compared with traditional tail-loading models. Future studies should include a tail-loading control group and direct physiological measurements, such as tail blood flow, local tissue inspection, blood pressure, and stress markers, to confirm whether the proposed model reduces tail-related vascular interference.”

Finally, although the vest system was designed to avoid interference with blood circulation and tail thermoregulation, future studies should include direct physiological assessments such as monitoring blood pressure, tail blood flow, or stress markers to further confirm that the apparatus does not induce undesirable hemodynamic effects, even though without any arterial occlusion the effect on this variable is obviously negligible. Considering these limitations in future studies will further strengthen the validity and applicability of the vest-pulley resistance training model as a refined experimental tool for investigating resistance exercise in hypertensive rodents.

## Conclusion

This study demonstrates the feasibility and functional effectiveness of a resistance training model for rats using a pulley system combined with a weighted vest. The model allowed the application of progressive overload during ladder climbing exercise without the need for tail load attachment, thereby avoiding potential interference with caudal blood flow and thermoregulation. However, the absence of fiber cross-sectional area and anabolic signaling analyses prevents definitive conclusions regarding myofiber hypertrophy or the molecular mechanisms of muscle remodeling. The training protocol produced significant strength gains and increased FHL mass, indicating that the model is capable of inducing functional and morphometric adaptations. Gene expression analyses of the genes selected for this study did not reveal statistically significant differences between the groups and therefore do not support mechanistic conclusions. Further explorations of more targets may yield important results. However, the primary objective of this study was to validate the training model, and the functional and morphometric findings support the pulley–vest system as a viable, refined, and reproducible experimental model for resistance training studies in rats, particularly in hypertensive models where preservation of tail circulation is important. This approach also contributes to animal welfare refinement by reducing the need for aversive stimuli commonly used in traditional tail-loading models.

## Data Availability

The original contributions presented in the study are included in the paper. Further inquiries can be directed to the corresponding author.

## References

[CR1] Al-Sarraf H, Mouihate A (2022) Muscle Hypertrophy in a Newly Developed Resistance Exercise Model for Rats. Front Physiol 13. 10.3389/fphys.2022.851789 PMC9136173 35634153

[CR2] Alway SE, Winchester PK, Davis ME, Gonyea WJ (1989) Regionalized adaptations and muscle fiber proliferation in stretch-induced enlargement. https://www.physiology.org/journal/jappl 10.1152/jappl.1989.66.2.771 2523374

[CR3] Amano Y, Nonaka Y, Takeda R, Kano Y, Hoshino D (2020) Effects of electrical stimulation-induced resistance exercise training on white and brown adipose tissues and plasma meteorin-like concentration in rats. Physiological Rep 8(16). 10.14814/phy2.14540 PMC7435032 32812347

[CR4] Aparicio VA, Nebot E, Porres JM, Ortega FB, Heredia JM, López-Jurado M, Ramírez PA (2011) Effects of high-whey-protein intake and resistance training on renal, bone and metabolic parameters in rats. Br J Nutr 105(6):836–845. 10.1017/S0007114510004393 21059282

[CR5] Armstrong RB, Phelps RO (1984) Muscle fiber type composition of the rat hindlimb. Am J Anat 171(3):259–272. 10.1002/aja.1001710303 6517030

[CR6] Baar K, Esser K (1999) Phosphorylation of p70(S6k) correlates with increased skeletal muscle mass following resistance exercise. Am J Physiol 6(1):C120–127. 10.1152/ajpcell.1999.276.1.C120 9886927

[CR7] Bodine SC, Baehr LM (2014) Skeletal muscle atrophy and the E3 ubiquitin ligases MuRF1 and MAFbx/atrogin-1. Am J Physiol Endocrinol Metab 307:469–484. 10.1152/ajpendo.00204.2014.-Muscle PMC4166716 25096180

[CR8] Caiozzo VJ (2002) Plasticity of skeletal muscle phenotype: Mechanical consequences. Muscle and Nerve 26(6):740–768. 10.1002/mus.10271 12451599

[CR9] Cardoso Cassilhas R, Teodoro Reis I, Venâncio D, Fernandes J, Tufik S, de Túlio M (2013a) Animal model for progressive resistance exercise: a detailed description of model and its implications for basic research in exercise 19(1)

[CR10] Cardoso Cassilhas R, Teodoro Reis I, Venâncio D, Fernandes J, Tufik S, de Túlio M (2013b) Animal model for progressive resistance exercise: a detailed description of model and its implications for basic research in exercise 19(1)

[CR11] Dias LG, Reis CHO, Dos Santos L, Krause Neto W, Lima-Leopoldo AP, Baker JS, Leopoldo AS, Bocalini DS (2024) Strength training improves heart function, collagen and strength in rats with heart failure. J Physiol Sci 74(1):10. 10.1186/s12576-024-00899-3 38365576 PMC10873996

[CR12] Feng M, Whitesall S, Zhang Y, Beibel M, D’Alecy L, DiPetrillo K (2008) Validation of volume-pressure recording tail-cuff blood pressure measurements. Am J Hypertens 21(12):1288–1291. 10.1038/ajh.2008.301 18846043

[CR13] Haizlip KM, Harrison BC, Leinwand LA (2015) Sex-based differences in skeletal muscle kinetics and fiber-type composition. In *Physiology* 30(1), pp. 30–39. American Physiological Society. 10.1152/physiol.00024.2014 PMC4285578 25559153

[CR14] Haun CT, Vann CG, Osburn SC, Mumford PW, Roberson PA, Romero MA, Fox CD, Johnson CA, Parry HA, Kavazis AN, Moon JR, Badisa VLD, Mwashote BM, Ibeanusi V, Young KC, Roberts MD (2019a) Muscle fiber hypertrophy in response to 6 weeks of high-volume resistance training in trained young men is largely attributed to sarcoplasmic hypertrophy. PLoS ONE 14(6). 10.1371/journal.pone.0215267 PMC6550381 31166954

[CR15] Haun CT, Vann CG, Osburn SC, Mumford PW, Roberson PA, Romero MA, Fox CD, Johnson CA, Parry HA, Kavazis AN, Moon JR, Badisa VLD, Mwashote BM, Ibeanusi V, Young KC, Roberts MD (2019b) Muscle fiber hypertrophy in response to 6 weeks of high-volume resistance training in trained young men is largely attributed to sarcoplasmic hypertrophy. PLoS ONE 14(6). 10.1371/journal.pone.0215267 PMC6550381 31166954

[CR16] Henselmans M, Vårvik FT, Izquierdo M (2026) The Effect of Carbohydrate Intake on Muscle Hypertrophy: A Systematic Review and Meta-analysis. Sports Med. 10.1007/s40279-025-02341-z 41712097 PMC13018098

[CR17] Hinks A, Jacob K, Mashouri P, Medak KD, Franchi MV, Wright DC, Brown SHM, Power GA (2022) Influence of weighted downhill running training on serial sarcomere number and work loop performance in the rat soleus. Biology Open 11(7). 10.1242/bio.059491 PMC9346294 35876382

[CR18] Hornberger TA, Farrar RP (2004) Physiological Hypertrophy of the FHL Muscle Following 8 Weeks of Progressive Resistance Exercise in the Rat. In Can. J. Appl. Physiol. Downloaded from www.nrcresearchpress.com by TEXAS STATE UNIV on 4(13). https://www.nrcresearchpress.com 10.1139/h04-002 15001801

[CR19] Júlio Chingui L, Padovan Braquinho R, Theresa Munhoz Severi M, Alberto da Silva C, Chingui RSão, Sebastião LJ (2008) Comportamento quimiometabólico do músculo sóleo na fase aguda da imobilização articular Chemical metabolic behaviour of the soleus muscle during the acute phase of joint immobilisation ENDEREÇO PARA CORRESPONDÊNCIA. 194 Fisioter Pesq 15(2):194–203

[CR20] Klitgaard H (1988) A model for quantitative strength training of hindlimb muscles of the rat. https://www.physiology.org/journal/jappl 10.1152/jappl.1988.64.4.1740 3379005

[CR21] Koopmans PJ, Williams-Frey TD, Zwetsloot KA (2024) Stuart has got the PoWeR! Skeletal muscle adaptations to a novel heavy progressive weighted wheel running exercise model in C57BL/6 mice. Exp Physiol 109(2):271–282. 10.1113/EP091494 37974360 PMC10988744

[CR22] Kraemer WJ, Ratamess NA (2004) Fundamentals of Resistance Training: Progression and Exercise Prescription. Medicine and Science in Sports and Exercise 36(4):674–688. 10.1249/01.MSS.0000121945.36635.61 15064596

[CR23] Krause Neto W, Silva W, Oliveira T, Vilas Boas A, Ciena A, Caperuto ÉC, Gama EF (2024) Ladder-based resistance training with the progression of training load altered the tibial nerve ultrastructure and muscle fiber area without altering the morphology of the postsynaptic compartment. Frontiers in Physiology, 15. 10.3389/fphys.2024.1371839 PMC11061484 38694209

[CR24] Kwon I (2025) Ladder-climbing resistance training stimulates upper limb flexor digitorum profundus muscle hypertrophy in rats. Phys Activity Nutr 29(3):23–32. 10.20463/pan.2025.0022 PMC12530985 41093307

[CR25] Liu D, Sartor MA, Nader GA, Gutmann L, Treutelaar MK, Pistilli EE, IglayReger HB, Burant CF, Hoffman EP, Gordon PM (2010) Skeletal muscle gene expression in response to resistance exercise: Sex specific regulation. BMC Genomics 11(1). 10.1186/1471-2164-11-659 PMC3091777 21106073

[CR26] Łochynski D, Kaczmarek D, Mrówczynski W, Warchoł W, Majerczak J, Karasinski J, Korostynski M, Zoladz JA, Celichowski J (2016) Contractile properties of motor units and expression of myosin heavy chain isoforms in rat fast-type muscle after volitional weight-lifting training. J Appl Physiol 121(4):858–869. 10.1152/japplphysiol.00330.2016 27539495

[CR27] Luciano TF, Marques SO, Pieri BL, De Souza DR, Araújo LV, Nesi RT, Scheffer DL, Comin VH, Pinho RA, Muller AP, de Souza CT (2017) Responses of skeletal muscle hypertrophy in wistar rats to different resistance exercise models. Physiol Res 66(2):317–323. 10.33549/physiolres.933256 27982685

[CR28] Neto WK, Ciena AP, Anaruma CA, Neto K, De Assis Silva W, Ciena WP, A., Gama F (2016) Vertical Climbing for Rodent Resistance Training: a Discussion about Training Parameters. Int J Sports Sci 6(1A):36–49. 10.5923/s.sports.201601.07

[CR29] Neves RVP, Souza MK, Passos CS, Bacurau RFP, Simoes HG, Prestes J, Boim MA, Câmara NOS, Franco M do C. P., Moraes MR (2016) Resistance training in spontaneously hypertensive rats with severe hypertension. Arquivos Brasileiros de Cardiologia 106(3):201–209. 10.5935/abc.20160019 PMC4811275 26840054

[CR30] Nicastro H, Zanchi NE, Da Luz CR, Chaves DFS, Lancha AH (2012) An experimental model for resistance exercise in rodents. J Biomed Biotechnol 2012. 10.1155/2012/457065 PMC3303681 22496606

[CR31] Nunes EA, Stokes T, McKendry J, Currier BS, Phillips SM (2022) Disuse-induced skeletal muscle atrophy in disease and nondisease states in humans: mechanisms, prevention, and recovery strategies. Am J Physiol Cell Physiol 322(6):C1068–C1084. 10.1152/ajpcell.00425.2021 35476500

[CR32] Rand RP, Burton AC, Ing T (1965) THE TAIL OF THE RAT, IN TEMPERATURE REGULATION AND ACCLIMATIZATION. Can J Physiol Pharmacol 43:257–267. 10.1139/y65-025 14329334

[CR33] Roberts MD, Haun CT, Vann CG, Osburn SC, Young KC (2020) Sarcoplasmic Hypertrophy in Skeletal Muscle: A Scientific Unicorn or Resistance Training Adaptation? In Frontiers in Physiology (Vol. 11). Frontiers Media S.A. 10.3389/fphys.2020.00816 PMC7372125 32760293

[CR34] Schiaffino S, Reggiani C (2011) Fiber Types In Mammalian Skeletal Muscles. Physiol Rev 91:1447–1531. 10.1152/physrev.00031.2010.-Mammalian 22013216

[CR35] Shiose K, Takahashi H, Yamada Y (2023) Muscle Glycogen Assessment and Relationship with Body Hydration Status: A Narrative Review. In Nutrients 15(1). MDPI. 10.3390/nu15010155 PMC9823884 36615811

[CR36] Soares LL, Leite LB, Ervilha LOG, da Silva BAF, de Freitas MO, Portes AMO, Rezende LMT, Drummond FR, Carneiro-Junior MA, Neves MM, Reis ECC, Natali AJ (2022) Resistance Exercise Training Mitigates Left Ventricular Dysfunctions in Pulmonary Artery Hypertension Model. Arquivos brasileiros de cardiologia 119(4):574–584. 10.36660/abc.20210681 36074480 PMC9563884

[CR37] Strickland JC, Smith MA (2016) Animal models of resistance exercise and their application to neuroscience research. J Neurosci Methods 273:191–200. 10.1016/j.jneumeth.2016.08.003. Elsevier B.V. PMC5075509 27498037

[CR38] Tamaki (1992) tamaki1992. Medicine Science in Sports and Exercise

[CR39] Wilde E, Aubdool AA, Thakore P, Baldissera L, Alawi KM, Keeble J, Nandi M, Brain SD (2017) Tail-cuff technique and its influence on central blood pressure in the mouse. J Am Heart Association 6(6). 10.1161/JAHA.116.005204 PMC5669161 28655735

